# High T-cell immune activation and immune exhaustion among individuals with suboptimal CD4 recovery after 4 years of antiretroviral therapy in an African cohort

**DOI:** 10.1186/1471-2334-11-43

**Published:** 2011-02-08

**Authors:** Damalie Nakanjako, Isaac Ssewanyana, Harriet Mayanja-Kizza, Agnes Kiragga, Robert Colebunders, Yukari C Manabe, Rose Nabatanzi, Moses R Kamya, Huyen Cao

**Affiliations:** 1 Department of Medicine, Makerere University School of Medicine, Kampala, Uganda; 2 Infectious Diseases Institute, Makerere University School of Medicine, Kampala, Uganda; 3 Joint Clinical Research Center, Kampala, Uganda; 4 California Department of Public Health, Richmond, California 94804, USA; 5 Institute of Tropical Medicine, Department of Clinical sciences, HIV/STD Unit, Antwerp, Belgium; 6 Department of Epidemiology and Social Sciences, University of Antwerp, Antwerp, Belgium

## Abstract

**Background:**

Antiretroviral therapy (ART) partially corrects immune dysfunction associated with HIV infection. The levels of T-cell immune activation and exhaustion after long-term, suppressive ART and their correlation with CD4 T-cell count reconstitution among ART-treated patients in African cohorts have not been extensively evaluated.

**Methods:**

T-cell activation (CD38+HLA-DR+) and immune exhaustion (PD-1+) were measured in a prospective cohort of patients initiated on ART; 128 patient samples were evaluated and subcategorized by CD4 reconstitution after long-term suppressive treatment: Suboptimal [median CD4 count increase 129 (-43-199) cells/μl], N = 34 ], optimal [282 (200-415) cells/μl, N = 64] and super-optimal [528 (416-878) cells/μl, N = 30].

**Results:**

Both CD4+ and CD8 T-cell activation was significantly higher among suboptimal CD4 T-cell responders compared to super-optimal responders. In a multivariate model, CD4+CD38+HLADR+ T-cells were associated with suboptimal CD4 reconstitution [AOR, 5.7 (95% CI, 1.4-23, *P *= 0.014)]. T-cell exhaustion (CD4+PD1+ and CD8+PD1+) was higher among suboptimal relative to optimal (*P *< 0.001) and super-optimal responders (P < 0.001). T-cell exhaustion was significantly associated with suboptimal responders [AOR, 1.5 (95%CI, 1.1-2.1), *P *= 0.022].

**Conclusion:**

T-cell activation and exhaustion persist among HIV-infected patients despite long-term, sustained HIV-RNA viral suppression. These immune abnormalities were associated with suboptimal CD4 reconstitution and their regulation may modify immune recovery among suboptimal responders to ART.

## Background

Although antiretroviral therapy (ART) partially corrects immune dysfunction associated with HIV infection, abnormalities of immune activation markers persist in many patients [1-3]. ART reduces the levels of T-cell activation by mechanisms independent of viral load [1-3]. Nevertheless, immune activation (as measured by expression of HLA-DR and CD38 on monocytes and T-cells) remained significantly higher among HIV-infected patients relative to the HIV-negative controls one year after viral suppression [4].

Up to 40% of individuals receiving ART have suboptimal CD4 T-cell recovery despite sustained HIV-RNA viral suppression [5-7]. HIV-RNA viremia and T-cell immune activation have been previously shown to be strong predictors of HIV disease progression [8]. In contrast, there is conflicting data on the influence of T-cell activation on CD4 T-cell recovery among patients on successful ART [9,10]. Furthermore, there is limited data on the influence of immune activation on CD4 count and functional recovery with sustained HIV-RNA suppression in African populations [11,12], where endemic co-infections may dampen the immune response [13].

Increased T-cell apoptosis has been proposed as a mechanism for unsatisfactory immune recovery [7]. PD-1 expression is increased during HIV-1 infection [14] and negatively regulates T-cell activity and can be measured by T-cell expression of Programmed Death-1 (PD-1) marker, an inducible molecule that is expressed on all lymphoid cells that are highly susceptible to apoptosis [15,16]. This study reports the levels of T-cell immune activation and T-cell exhaustion among adult Ugandans with sustained HIV-RNA viral suppression after 4 years of ART.

## Methods

### Study setting and participants

Between April, 2004 and April, 2005, 559 consecutive ART-naïve HIV-infected patients, were initiated on ART and enrolled into the Infectious Diseases Institute (IDI) prospective observational research cohort as previously described [17]. Patients were initiated on first-line ART at CD4 counts ≤ 200 cells/μl according to Ugandan guidelines for ART initiation at the time. Drugs were provided through the Global Fund (a generic combined formulation of stavudine [d4T, lamivudine [3TC], and nevirapine[NVP] and the US President's Emergency Plan for AIDS Relief (a combined formulation of zidovudine [ZDV] and 3TC plus efavirenz [EFZ] or nevirapine [NVP]. Patients with toxicity to ZDV were changed to tenofovir [TDF]. All patients received cotrimoxazole (or dapsone) prophylaxis according to the national policy to provide cotrimoxazole to all people living with HIV (PLHIV). Adherence to ART was encouraged by at least 3 individual and group counseling sessions. Patients were reviewed monthly by the study physicians that evaluated among others; adherence to medication, toxicities and acute infections. HIV RNA viral loads, complete blood counts and CD4 lymphocyte counts were measured 6 monthly intervals.

**Figure 1:**
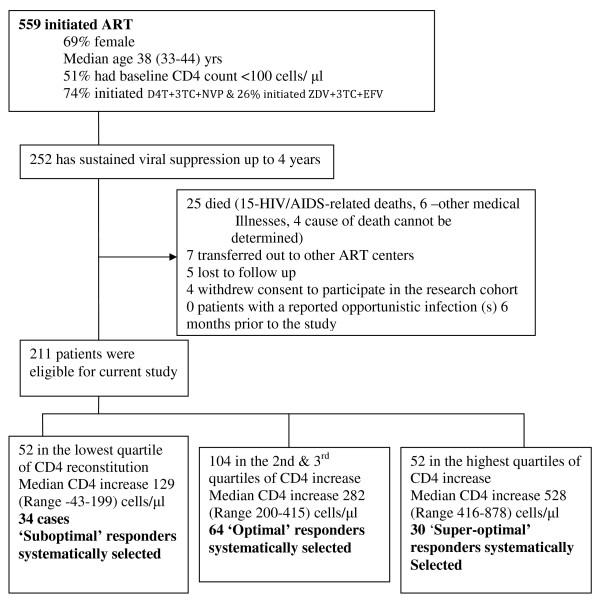
**Profile of patients on antiretroviral therapy within the Infectious Disease research cohort**.

After 4 years of follow up on ART, 252/559 (45%) patients had sustained HIV-RNA viral suppression. Of these, 41 were excluded due to the following reasons; death (n = 25), lost to follow-up (n = 5), voluntary request to transfer to and voluntary termination from the study (n = 11). We excluded patients that had an opportunistic infection in the previous 6 months. Up to 128 patients with chronic HIV infection successfully suppressed on ART with HIV RNA levels < 400 copies/ml for 4 years were evaluated: 34 patients (cases) with suboptimal CD4 reconstitution, 64 with optimal CD4 reconstitution (controls) and 30 with super-optimal CD4 reconstitution (controls); see Figure 1.

### Definition of suboptimal CD4 reconstitution

**Various definitions have **been used to describe suboptimal CD4 reconstitution following ART including the magnitude of the CD4 cell increase [18]. Kaufmann et al defined suboptimal CD4 reconstitution an absolute CD4 count < 500 cells/μl after 5 years of sustained viral loads < 1000 copies [19]. However, less than one-third of all the patients in our African study cohort had attained an absolute CD4 count ≥ 500 cells/μl. Therefore we used a cohort-specific definition of suboptimal CD4 reconstitution in order to consider the whole spectrum of CD4 recovery. The magnitude of CD4 increase (difference between absolute CD4 counts at baseline and absolute CD4 counts after 4 years of ART), for the 211 patients with sustained HIV-RNA viral suppression, was grouped into 4 quartiles. Cases of **'Suboptimal CD4 reconstitution' **included patients within the lowest quartile and the controls with **'Optimal CD4 reconstitution' **included patients within the two middle quartiles while the controls with **'Super-optimal CD4 reconstitution' **included patients within the highest quartile of CD4 increase (see Figure 1). Written informed consent was obtained from all participants. This study was approved by national institutional review board; the Uganda National Council of Science and Technology. The immunology assays were performed at the cytotoxic T lymphocyte laboratory (CTL) at the Joint clinical research center (JCRC), Kampala, Uganda.

### PBMC separation

Fifty mls of whole blood, collected in ACD-A bottles, was processed for PBMC separation within 4 hours of collection. PBMCs were separated by Ficoll-Hypaque density configuration, washed and re-suspended in phosphate buffer saline (PBS) containing heat inactivated fetal calf serum (FCS). PBMCs were frozen and stored in FBS with 10% dimethyl sulfoxide (DMSO), in liquid nitrogen until assay time.

### Cell surface staining for immune profile

PBMC were thawed and rested overnight before surface staining. PBMC viability was 90% and above. Surface staining for immune profile was done in two panels by incubating with the following antibodies; CD3 APC, CD4 PerCP-Cy5.5, HLA-DR FITC and CD38 PE (BD Biosciences San Jose, CA) for immune activation and CD3 FITC, CD4 PerCP-Cy5.5, PD-1 APC and CD127 PE (BD Biosciences San Jose, CA) for cell function/exhaustion. The florescence signals were measured by 4-color flow Facscalibur

(BD Biosciences San Jose, CA). In general, at least 50,000 events in the CD3-positive gate were collected. Gating was standardized and set using fluorescence minus one controls (FMOs) for HLADR, CD38 and PD-1. Data were analyzed using FLOWJO software (TreeStar, San Carlos, CA). Immune activation was defined as the percent of CD38+ HLA DR+ T-cells. PD-1 level was defined as the percent expression of PD-1 APC CD3^+ ^CD8^+ ^(or CD4+) T-cells, respectively.

### Co-infections

All participants were tested for concurrent acute infections that are associated with immune activation. Among other tests, patients were tested for hepatitis B surface antigen (hepatitis B surface antigen version 3, Murex Biotech LTD, Darford, UK) and intestinal helminthes (using stool microscopy for parasites, ova and cysts and modified ZN stain for cryptosporidia infestations) as well as C-reactive protein levels, a biomarker for acute non-specific acute infection.

### Statistical analysis

We compared percentages of activated (CD38+HLADR+) T-cells between patients with suboptimal, optimal and super-optimal CD4 reconstitution after 4 years of ART with plasma HIV-RNA levels below 400 copies/ml. Chi square and Kruskal-Wallis tests were used for unadjusted comparisons between the three groups for categorical and continuous variables respectively. Wilcoxon rank sum test was used compare immunological and clinical parameters among suboptimal and super-optimal responders. Logistic regression was used to determine relationship between T-cell activation and CD4 reconstitution. All factors that were significant predictors of suboptimal CD4 reconstitution in unadjusted analyses (P < .10) were included in the multivariate model in a stepwise manner.

## Results

### Characteristics of participants

Overall, 128 individuals with sustained HIV-RNA viral loads < 400 copies/ml after 4 years of ART were evaluated: 34 patients with 'suboptimal CD4 reconstitution', 64 with 'optimal CD4 reconstitution' and 30 with 'super-optimal CD4 reconstitution'. Median CD4 count increase (difference between baseline and 4 years of ART) was 129 (-43-199), 282 (200-415) and 528 (416-878) cells/μl for the suboptimal, optimal and super-optimal respectively (see Figure 1). Baseline clinical characteristics were similar among the three patient categories both at baseline and after 4 years of ART except CD4 counts that were significantly different after 4 years of ART (*P *< 0.001); see Table 1.

**Table 1:** Characteristics of 128 patients with sustained viral suppression after 4 years of antiretroviral therapy at the Infectious Diseases Institute research cohort∏

	Sub-optimal CD4 reconstitution (Cases) N = 34	Optimal CD4 reconstitution (Controls) N = 64	Super-optimal CD4 reconstitution (Controls) N = 30	P value*
**At baseline**				
Age (yrs) [median (IQR)]	36 (31-42)	33 (30-38)	35 (31-43)	0.509
Female gender [n (%)]	21 (62)	49 (77)	26 (87)	0.066
BMI [median (IQR)]	20 (19-23)	20 (19-23)	20 (19-23)	0.935
CD4 cells/μl [median (IQR)]	111 (62-151)	105 (40-176)	115 (97-185)	0.447
< 100 cells/μl [n(%)]	20 (59)	35 (55)	15 (30)	0.779
ART regimen				
D4T-3TC-NVP/EFZ [n (%)]	17 (50)	43 (67)	22 (73)	
ZDV-3TC-NVP/EFZ [n (%)]	17 (50)	21 (33)	8 (27)	0.116
Hemoglobin [ median (IQR)]	12 (11-13)	11 (10-13)	11 (10-13)	0.473
**After 4 years of ART**				
CD4 cells/μl [median (IQR)]	220 (183-248)	407 (334-456)	985 (438-1290)	< 0.001
< 350 cells/μl [n(%)]	34 (100)	19 (30)	0 (0)	< 0.001
BMI [median (IQR)]	21 (19-25)	22 (20-24)	22 (20-24)	0.062
Hemoglobin [ median (IQR)]	14 (12-15)	13 (12-14)	13 (12-14)	0.969
Hepatitis B positive [n]	1	5	1	0.033
ART regimen				
D4T-3TC-NVP/EFZ [n (%)]	13 (38)	41 (64)	17 (57)	
ZDV-3TC-NVP/EFZ [n (%)]	21 (62)	18 (28)	13 (43)	
TDF-3TC-NVP/EFZ [n (%)]	0 (0)	5 (8)	0 (0)	0.008

*Chi square test was used for categorical variables and Kruskal-Wallis test was used for the continuous variables.

∏All patients initiated antiretroviral therapy at CD4 200 cells/μl and below. The magnitude of CD4 increase from the baseline counts was grouped into 4 quartiles; 'Suboptimal CD4 reconstitution' includes patients that lie in the lowest quartile, 'Optimal CD4 reconstitution' includes a sample of patients that lie in the two middle quartiles and 'Super-optimal CD4 reconstitution' includes patients that lie in the highest quartile of CD4 increase.

### T-cell activation

**Figure 2:**
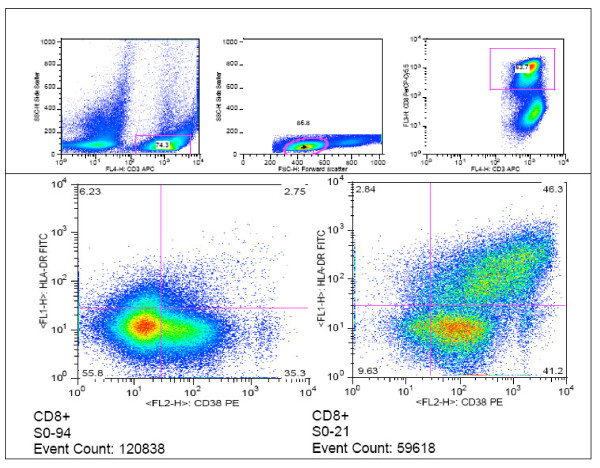
**Flow cytometry analysis of CD38+HLADR+ CD8 T cells**. The upper panel shows the gating strategy for co-expression of CD38 and HLADR (immune activation) by CD8 T cells. The lower panel shows a 'super responder' with a typically low proportion of activated CD8 T cells (left) and a 'suboptimal responder' with a typically high proportion of activated CD8 T cells.

**Figure 3:**
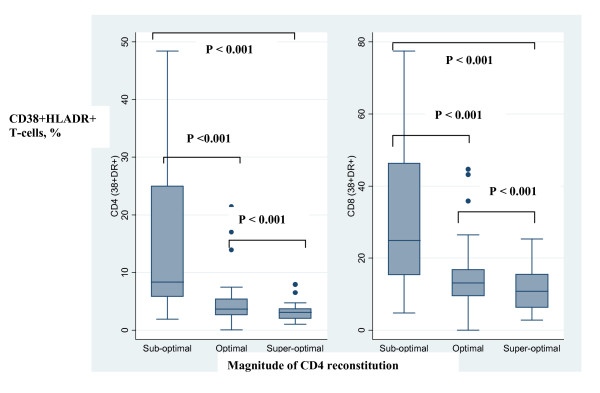
**T-cell activation and magnitude of CD4 count increase among HIV-infected patients after 4 years of antiretroviral therapy and sustained HIV-RNA viral suppression**. Immune activation, was measured by co-expression of HLADR and CD38 (CD38+HLADR+). The percentages of activated CD4 (CD438+HLADR+) and CD8 (CD8 CD38+ HLADR+) T-cells were plotted against CD4 count reconstitution. Immune activation of CD4 and CD8 T-cells was higher among patients with suboptimal CD4 reconstitution (patients that lie in the lowest quartile of CD4 increase) relative to the super-optimal responders (patients that lie in the highest quartile of CD4 increase) P = 0.001. The boxes span the 25^th ^and 75^th ^percentile values, the error bars span the 10^th ^and 90^th ^percentile values, and the dots represent individual observations above the 90^th ^percentile values.

T-cell activation was measured by co-expression CD38 and HLADR (CD4+ CD38+HLADR+ and CD8+CD38+HLADR+) on the T cell surface (see Figure 2). CD4 and CD8 T-cell activation was significantly higher among 'suboptimal' responders compared with the 'optimal' *(p < 0.001) *and 'super-optimal' responders *(p < 0.001); *see Figure 3. In addition, CD4+CD38+HLADR+ T-cells were independent predictors of suboptimal CD4 reconstitution [Adjusted odds ratio, AOR, 5.7 (95% confidence interval, 95%CI, 1.4-23, P = 0.014)].

### T-cell exhaustion

**Figure 4:**
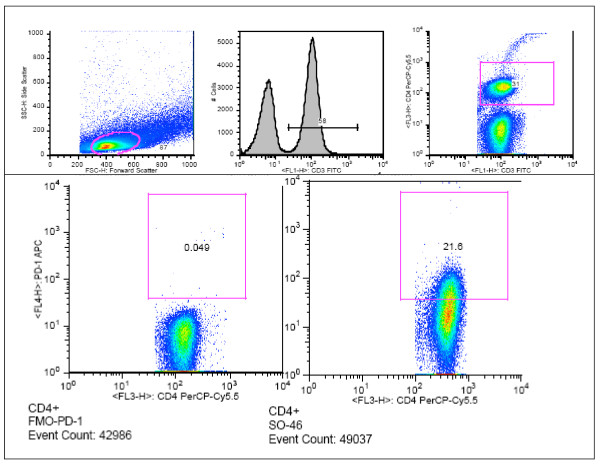
**Flow cytometry analysis of PD1+ CD4 T cells for an individual with suboptimal CD4 reconstitution**. The upper panel shows the gating strategy for the PD1+ T cells that are marked for apoptosis. The lower panel shows the fluorescence minus one control (FMO) for PD1 (left) and a typically high proportion of PD1+ cells (right).

**Figure 5:**
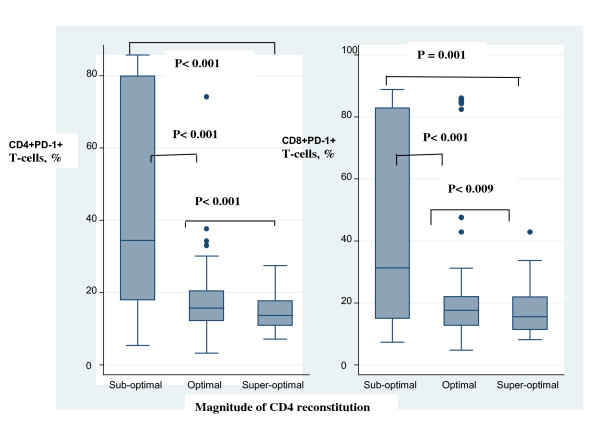
**T-cell exhaustion among HIV-infected patients after 4 years of antiretroviral therapy with sustained HIV-RNA viral suppression**. T-cell exhaustion was measured by expression of programmed cell death 1 (PD-1) by CD4 and CD8 cells (CD4 PD1+ and CD8 PD1+). The percentages of CD4 PD1+ and CD8 PD1+ T-cells were plotted against CD4 count reconstitution. Exhaustion of CD4 and CD8 T-cells was significantly higher among patients with suboptimal CD4 reconstitution (patients that lie within the lowest quartile of CD4 increase) relative to optimal responders (patients that lie within the middle quartiles of CD4 increase and super-optimal responders (patients that lie within the highest quartile of CD4 increase) P = 0.001. The boxes span the 25^th ^and 75^th ^percentile values, the error bars span the 10^th ^and 90^th ^percentile values, and the dots represent individual observations above the 90^th ^percentile values.

Immune exhaustion was measured by expression of PD-1 on CD4 (CD4+PD1+) and CD8 (CD8+PD1+) T-cells (see Figure 4). PD-1 expression was significantly higher among 'suboptimal responders' compared with the optimal *(p < 0.001) *and 'super-optimal' *(p < 0.001) *responders (see Figure 5). CD4+PD1+ remained significantly higher among suboptimal responders at multivariate analysis [AOR, 1.5 (95%CI 1.1-2.1), *P *= 0.022]; see Table 2.

**Table 2:** T-cell immune activation, cell exhaustion and peripheral blood parameters among patients with and without suboptimal CD4 reconstitution after 4 years of antiretroviral therapy.

Parameters measured after 4 years of ART	Suboptimal CD4 reconstitution (Cases) N = 34	Super-Optimal CD4 reconstitution (Controls) N = 30	P-value *	Adjusted OR (95% CI)	P-value
**Immune activation**					
CD4 HLADR+ CD38+ [Median (IQR)]	7.9 (4.9-16.4)	2.8 (2.0-3.6)	< 0.001	5.7 (1.4-23.0)	0.014
CD8 HLADR+ CD38+ [Median (IQR)]	22.5 (15.3-44.9)	10.3 96.3-15.1)	< 0.001	0.8 (0.6-1.1)	0.107
**Cell exhaustion**					
CD4 PD1+ [Median (IQR)]	34.3 (17.6-78.8)	7.3 (4.7-9.4)	< 0.001	1.5 (1.1-2.1)	0.022
CD8 PD1+ [Median (IQR)]	30.6 (13.4-82.9)	7.9 (6.2-9.5)	< 0.001	1.2 (0.9-1.5)	0.130
**Peripheral blood parameters**					
Hemoglobin (mg/dl) [Median (IQR)]	13 (12-14)	14 (12-15)	0.293		
MCV (fl) [Median (IQR)]	102 (96-112)	109 (98-112)	0.264		
Total white blood cell count [Median (IQR)]	3530 (3000-4050)	5050 (4240-5720)	0.003		
Lymphocytes % [Median (IQR)]	37 (30-46)	45 (36-51)	0.278		
Neutrophils % [Median (IQR)]	45 (40-59)	42 (38-50)	0.820		
Eosinophils % [Median (IQR)]	4 (1-8)	2 (1-3)	0.012		
Platelet count nx10^3 ^[Median (IQR)]	235 (205-284)	292 (214-318)	0.359		

*Wilcoxon rank-sum test, ¥ Chi square, MCV, mean corpuscular volume

### Peripheral blood parameters

The total white blood cell count and eosinophil percentage were significantly lower among suboptimal responders relative to super-optimal responders; *P*-values; <0.003, and 0.012 respectively (see Table 2). However, at multivariate analysis these parameters were not significant predictors of suboptimal CD4 reconstitution (data not shown).

### Co-infections

All participants were tested for concurrent acute infections that are associated with immune activation. Of the 128 patients with sustained viral suppression, 7(6%) were positive for hepatitis B surface antigen, only 13 (10%) had a C-reactive protein level above 6 mg/dl. No intestinal parasitic infection was observed in all participants using both stool microscopy for ova and parasites and modified ZN stain.

## Discussion

Immune activation, as defined by expression of HLA-DR and CD38 by T-cells typically normalizes several months following successful initiation of ART [20]. However, we describe high levels of immune activation in a cohort of HIV infected Ugandan adults four years following ART; a report that is consistent with several other studies from the Western cohorts [21,22]. Contrary to previous studies from the West [10], this study demonstrated that suboptimal CD4 reconstitution is strongly associated elevated T cell activation, regardless of initial clinical parameters. Various immunosuppressive agents have been investigated in the interest of "turning off" excessive immune activation [23,24] as an intervention to improve patients' responses to ART. Therefore down-regulation of immune activation is a potential strategy to optimize immune recovery among ART-treated patients with suboptimal CD4 reconstitution. Noteworthy, the study participants were started on ART at CD4 counts < 250 cells/μl. This is clearly severe immune suppression considering the growing evidence initiation of ART at higher CD4 counts yields better clinical and immunological outcomes [25,26]. This study adds evidence to the current evidence that early initiation of ART not only increases CD4 counts and survival [6,25-27], but also lowers the levels of T-cell activation and possibly improves T-cell function recovery. Therefore early targeted and aggressive intervention in the population with suboptimal immune recovery may be beneficial [28-30].

The high levels of T-cell immune-activation did not correlate with the presence of co-infections including tuberculosis, cryptococcal meningitis, Pneumocystis *iiroveci *pneumonia, toxoplasmosis, oro-esophageal candidiasis and malaria. This result is consistent with our previous report that AIDS-related events were no more among patients with and without suboptimal CD4 reconstitution [6]. Similarly, none of the patients was found to have intestinal helminthiasis despite living in a region where the infection is endemic. The authors attribute this to the fact that this was a selected population that receives comprehensive screening, treatment and prevention of co-infections that includes among others; cotrimoxazole prophylaxis, regular de-worming and a safe water vessel; all of which reduce the risk of the co-infections [31-34].

Expression of programmed cell death 1 (PD-1) was significantly up-regulated on T-cells of suboptimal responders relative to super-optimal responders. Significant difference in T-cell immune activation and exhaustion was observed despite undetectable viremia. This implies that ongoing viral replication may not be the driver of PD-1 expression as previously reported [35]. However, our results are in agreement with evidence that increased apoptosis and intrinsic T-cell death play a role in incomplete CD4 count recovery [7]. There is need for further studies to determine other potential drivers of both immune activation and PD-1 expression among HIV-infected patients on successful ART. Similarly, advances to regulate these immunological abnormalities may modify CD4 count recovery among ART-treated HIV-infected patients with suboptimal CD4 reconstitution.

Our study design did not permit determination of the causation of immune dysregulation. Although majority of available data suggests that immune activation is most likely a cause of the damaged immune system rather than a consequence [30], it is possible that lack of CD4 recovery could be the cause and not a consequence of the immune impairment [36]. We did not compare levels of immune activation among patients with and without viral suppression however there is already evidence that ART decreases immune activation levels over time [1,37].

## Conclusion

T-cell activation and exhaustion persist among HIV-infected patients despite long-term, sustained HIV-RNA viral suppression. These immune abnormalities were associated with suboptimal CD4 reconstitution and their regulation may modify immune recovery among ART-treated patients with suboptimal CD4 reconstitution despite sustained viral suppression.

## Competing interests

The authors declare that they have no competing interests.

## Authors' contributions

DN made substantial contribution to the conception, design, data collection, analysis and drafting of the manuscript. IS contributed to the immune assays, data analysis and interpretation. HMK contributed to the conception, design, data interpretation and revision of the manuscript. AK made substantial contribution to the study design and the statistical analysis. RC and YCM contributed to the conception, data interpretation and revision of the manuscript. RN contributed to the data collection and MRK contributed to the conception, design, data interpretation and revision of the manuscript. HC made substantial contribution to the conception, design, immune assays, data analysis, interpretation and revision of the manuscript. All authors read and approved the final manuscript.

## Pre-publication history

The pre-publication history for this paper can be accessed here:

http://www.biomedcentral.com/1471-2334/11/43/prepub
